# *Notch, Wnt, *and* Hedgehog * Pathways in Rhabdomyosarcoma: From Single Pathways to an Integrated Network

**DOI:** 10.1155/2012/695603

**Published:** 2012-03-11

**Authors:** Josep Roma, Anna Almazán-Moga, Josep Sánchez de Toledo, Soledad Gallego

**Affiliations:** ^1^Laboratori de Recerca Translacional en el Càncer Pediàtric, Hospital Universitari Vall d'Hebron, Universitat Autònoma de Barcelona, Pg Vall d'Hebron 119-129 08035, Spain; ^2^Departament d'Oncologia i Hematologia Pediàtriques, Hospital Universitari Vall d'Hebron, Universitat Autònoma de Barcelona, Pg Vall d'Hebron 119-129 08035, Spain

## Abstract

Rhabdomyosarcoma (RMS) is the most common type of soft tissue sarcoma in children. Regarding histopathological criteria, RMS can be divided into 2 main subtypes: embryonal and alveolar. These subtypes differ considerably in their clinical phenotype and molecular features. Abnormal regulation or mutation of signalling pathways that regulate normal embryonic development such as *Notch, Hedgehog,* and *Wnt* is a recurrent feature in tumorigenesis. Herein, the general features of each of the three pathways, their implication in cancer and particularly in RMS are reviewed. Finally, the cross-talking among these three pathways and the possibility of better understanding of the horizontal communication among them, leading to the development of more potent therapeutic approaches, are discussed.

## 1. Introduction

Rhabdomyosarcoma (RMS) is the most common type of soft tissue sarcoma in children. RMS can be divided into 2 main histopathological subtypes: embryonal and alveolar (ERMS and ARMS, resp.). These subtypes differ considerably in their clinical phenotype and molecular features. The prognosis of ERMS is more favourable than that of ARMS. From a molecular point of view, the majority of ARMS (80% to 85%) contain one of the reciprocal chromosomal translocations: either t(2;13) (q35;q14) or t(1;13)(p36;q14). These translocations generate the anomalous fusion genes *PAX3-FOXO1* and *PAX7-FOXO1*, respectively [1, 2]. The resulting chimerical proteins have potent transforming effects and are thought to inhibit myogenic differentiation. However, no characteristic translocations have been described in ERMS. The ERMS is typically characterised by loss of heterozygosity on the short arm of chromosome 11 (11p15.5) [3], and gains in chromosomes 2, 7, 8, 11, 12, 13, and 17 are also common in this subtype [4].

*Notch*, *Wnt,* and *Hedgehog* pathways are known to play critical roles in the development of pluricellular organisms. Knowledge of the oncogenic role (by mutation or deregulation) of these pathways has been widening in recent decades. In paediatric malignancies, evidence of the possible significance of these pathways in the promotion of oncogenic phenotype has been accumulating. Although the understanding of the roles played by these pathways in paediatric tumours is advancing, it is far from that of better known adult malignancies. 

## 2. *Notch* Signalling

*Notch* signalling plays a critical role in tissue development in organisms ranging from nematodes to mammals. The *notch* genes encode 4 highly conserved cell surface receptors that are activated by its ligands (*Delta* and *Jagged* in vertebrates). The *Notch* intracellular domain (*NICD*) is then proteolysed and released by the **γ*-secretase* complex and translocates to the nucleus where it binds to CSL transcription repressors, converting them into transcriptional activators. The paradigmatic targets of these transcription factors in vertebrates are the *HES* and *HEY* genes [5, 6].

### 2.1. *Notch* and Cancer

The oncogenic potential of the *Notch* pathway was first described in acute T-cell lymphoblastic leukaemia (T-ALL) in the late 1980's. In normal conditions, *Notch* signalling is necessary for correct maturation of T-cell progenitors; however, constitutive activation of the pathway leads to abnormal T-cell proliferation causing T-ALL [7]. An abnormal upregulation of the *Notch* pathway has also been reported in ovarian [8], breast [9], and other cancers (cervix, head and neck, endometrium, kidney, lung, pleural mesothelioma, malignant melanoma, Hodgkin's lymphoma, anaplastic large cell lymphomas, some acute myeloid leukaemias, and chronic B-cell lymphocytic leukaemia, among others) [10]. With respect to paediatric malignancies, *Notch* signalling appears to contribute essentially to osteosarcoma metastasis [11] and proliferation [12]; *Notch* signalling also promotes medulloblastoma cancer stem cell survival [13] and contributes to angiogenesis in neuroblastoma [14].

### 2.2. *Notch* and RMS

During normal muscle development, the *Notch* pathway is involved in satellite cell activation and in cell fate determination during postnatal myogenesis [15]. Activation of *Notch* pathway is known to inhibit myogenesis [16, 17]. However, the role of the pathway in RMS is barely known. Our group recently showed that the *Notch* pathway is widely and consistently activated in both ARMS and ERMS patients; a clear implication in the regulation of motility and invasiveness of ARMS and ERMS cells was also reported in the same work [18]. The existence of a wide range of pharmacological *Notch* inhibitors renders this pathway a promising therapeutic target in the fight against metastases; however, the cross-talk with other pathways such as *Hedgehog* and *Wnt*—as will be discussed below—may negatively influence the efficacy of therapeutic approaches if directed exclusively against one pathway.

## 3. *Hedgehog* Signalling

*Hedgehog* (*Hh*) signalling was first described in 1980 as a gene exerting a direct effect on embryonic development in *Drosophila *[19]. *Hedgehog* genes are considered to be key regulators of development in organisms ranging from the fruit fly to mammals, since they control multiple embryonic processes such as tissue patterning, proliferation and differentiation. *Hedgehog* signalling also plays important roles in adult organisms such as stem cell maintenance and tissue repair and regeneration. The 3 Hedgehog proteins present in mammals, Sonic (*SHh*), Indian (*IHh*), and Desert (*DHh*), need a maturation process to achieve their active forms. This maturation process implies autocatalytic cleavage of the protein to release its active N-terminal peptide, with subsequent N-palmitoylation and the formation of a C-terminal cholesterol adduct [20]. Mature *Hh* proteins are ligands of patched receptors (*Ptch1* and *Ptch2*). Ligand-free *Ptch* inhibits the activation of *Smoothened* (*Smo)* by an incompletely known mechanism. The hypothesis that one molecule of *Ptch* inhibits one molecule of *Smo* by direct binding has been ruled out. Instead, the existence of endogenous small molecules, gated by *Ptch*, which are able to modulate *Smo* activity, is currently under discussion [21]. Although the exact endogenous small molecule that modulates *Smo* activity has not been identified, sterol-like molecules have emerged as leading candidates [21]. In the absence of active *Smo* in the membrane, GLI family zinc finger proteins (*Gli1, Gli2, and Gli3*) in a complex with *SuFu* (suppressor of fused homolog) are proteosomically processed. Upon binding of a *Hedgehog* ligand, active *Smo* is detected in the membrane and prevents *Gli* proteasomal processing. *Gli* is then translocated to the nucleus where it binds to *Gli*-specific promoters. *Gli1* and *Gli2* mainly function as transcriptional activators, whereas *Gli3* exists in two forms, either as a full-length transcriptional activator (*Gli3A*) or an amino-terminal fragment that functions as a repressor (*Gli3R*). The three best known direct targets of the pathway are *Gli1*, *Ptch1,* and *Hhip*, genes of the pathway itself [21–23].

### 3.1. *Hedgehog* and Cancer

The *Hedgehog* pathway also has major implications in several cancers. Mutation or deregulation of the pathway may lead to tumorigenesis in a wide variety of tissues. The initial link between *Hedgehog* signalling and human cancers was established when mutations in human *PTCH1* were found to be associated with a rare hereditary disease called Gorlin's syndrome. Patients with Gorlin's syndrome have a high incidence of basal cell carcinoma, medulloblastoma, and rhabdomyosarcoma. The molecular origin of this syndrome is a constitutive activation of the *Hedgehog* pathway caused by mutations in the *PTCH1* gene [24, 25]. *Hedgehog* pathway alterations—mainly loss of function of *Ptch* and *SuFu* or activating mutations in *Smo*, *Hh,* or *Gli*—are thought to be oncogenic in a considerable number of other cancers (gliomas, breast, lung, prostate, ovarian, colon, and endometrial carcinomas, multiple myeloma, and chronic myeloid leukaemia, among others) [26].

### 3.2. *Hedgehog* and RMS

The role of *Hedgehog* signalling in the genesis of RMS was first described in the *Patched* knockout mouse by Hahn et al. in 1998 [27] who reported that mice heterozygous for *Ptch1* not only develop features consistent with Gorlin's syndrome, such as generalised overgrowth of the body and a variety of neural and skeletal abnormalities, but also have a high incidence of ERMS. Tumours in heterozygous *Ptch1* mice exhibited elevated transcript levels of *Gli1* and *Ptch1* itself, indicating that abnormal *Hedgehog* signalling may be common to the various tumours associated with Gorlin's syndrome [27]. The formation of RMS in *Ptch1-*mutant mice has been associated with the ability of tumour cells to resist apoptosis [28]. The role of epigenetic regulation of *Ptch1* expression seemed to be crucial in heterozygous *Ptch1* mice, since a combined treatment with the DNA-methyltransferase1 inhibitor 5-aza-2′deoxycytidine and the histone deacetylase inhibitor valproic acid efficiently prevented RMS and medulloblastoma formation in this model [29]. Currently, a consistent activation of the pathway is well established and generally accepted in RMS. A higher degree of *Hh* activation in ERMS and translocation-negative ARMS than in translocation-positive ARMS has also been reported [30]. In the same work, Zibat et al. analysed the pathway in a large cohort of RMS patients and hypothesised that the activation of the pathway confers poor prognosis in ERMS and translocation-negative ARMS and suggested an inverse correlation between *Hh* pathway activation and muscular differentiation [30]. Conversely, Pressey et al. stated recently that neither *GLI1* nor *PTCH1* mRNA transcripts in ERMS tumours correlated with survival or other clinical characteristics analysed [31]. Another recent work showed positive staining by immunohistochemistry of 78% of samples for *SHh*, 100% for *Ptch,* and 78% for *Gli1* in a panel of 18 RMS and reported, in disagreement with the work of Zibat et al., higher *Gli1* expression in alveolar than in embryonal subtypes [32]. On the other hand, several publications that combine *in vitro* and *in vivo* works with xenografted rhabdomyosarcoma models agree about the possibility to effectively reduce tumour growth by *Hh* pathway inhibition mediated by betulinic acid, GANT-61, and forskolin [33–35]. Although these treatments did not achieve total remission of the tumour, the significant reduction in tumour growth suggests that *Hh* signalling plays a leading role in RMS oncogenicity and that the pathway can be considered a potential molecular target for new treatment strategies in this neoplasia. In fact, derivatives of cyclopamine and other small molecular antagonists of *Smo* have recently entered clinical phase I and II trials for basal cell carcinoma, with encouraging results particularly for GDC-0449 [36, 37].

## 4. *Wnt* Signalling

The first description of the gene *Wingless* in Drosophila and the subsequent discovery of its orthologous genes in vertebrates laid the keystone of an evolutionary conserved signalling pathway now commonly referred to as the *Wnt *pathway. This pathway is involved in the establishment of the body axis at the earliest stages of embryogenesis and is also later required for development of many organs in organisms ranging from *C. elegans* to mammals. Two variants of the pathway, canonical and noncanonical, have been described. The *Wnt* canonical pathway is generally thought to regulate cell fate determination and the non-canonical one to control cell movement and tissue polarity [38].

In the canonical pathway and in the absence of *Wnt* ligands, *APC* and *Axin* bind to **β*-catenin* (the central actor in the canonical pathway) thereby permitting its *phosphorylation by casein kinase I alpha* (*CKI*α**) and *glycogen synthase kinase 3 beta* (*GSK3*β*)* and, sequentially, its polyubiquitination and proteosome-mediated degradation. The interaction of *Wnt* ligands with *Frizzled* receptor and *Lrp5/6* coreceptor inhibits the degradation of **β*-catenin* owing to the formation of *Frizzled-Dishevelled* and *Lrp5/6-Axin-FRAT* complexes and the inactivation of *GSK3*β** via *Dishevelled (Dvl)*. Stable **β*-Catenin* is translocated to the nucleus where it binds to *T-cell factor/lymphoid enhancer factor* (*TCF/LEF*) and also to *Legless family docking proteins *(*BCL9 and BCL9L*) associated with *PYGO* family coactivators, thereby promoting transcription of the target genes of this pathway: *FGF20*, *DKK1*, *WISP1*, *MYC,* and *CCDN1*, among others [38]. The non-canonical pathway is also initiated by *Wnt* ligands (exemplified by *Wnt5a* and *Wnt11*) and *Frizzled* receptors; however, while the canonical pathway leads to **β*-catenin* and *TCF/LEF*-mediated gene expression, non-canonical *Wnt* signalling is mainly mediated by activation of *PKC* and *JNK* [39, 40].

### 4.1. *Wnt* and Cancer

Anomalous activation of the *Wnt* pathway has been reported in several adult cancers such as non-small-cell lung cancer, colorectal carcinoma, prostate cancer, and breast cancer among others. Deregulation of the *Wnt* pathway in carcinogenesis is often attributed to a mutation in the **β*1-Catenin* gene (*CTNNB1*). Oncogenic involvement of the *Wnt* pathway has also been shown in different embryonal tumours, such as hepatoblastoma, nephroblastoma (Wilms' tumour), pancreatoblastoma, and medulloblastoma. Up to 75% of hepatoblastomas and 15% of Wilms' tumours display *CTNNB1* mutations, approximately half of which affect exon 3 [41–43]. Other cancer studies revealed a downregulation of the tumour suppressor *Wnt5a*, related to the non-canonical pathway, the deletion or reduced expression of which can occur in several cancers including sarcomas [44–46].

### 4.2. *Wnt* and RMS

Very few works on the role of *Wnt* pathway in RMS have been published. Soglio et al. concluded that there was no evidence of **β*1-Catenin* mutation in the genesis of RMS and that this protein did not constitute a useful marker for distinguishing between ERMS and ARMS [47]. Analysis of the *CTNNB1* gene sequence in 8 ERMS and 3 ARMS revealed no **β*1-Catenin* mutations in this patient cohort. In the same work, an immunohistochemical study of **β*1-Catenin* location showed cytoplasmatic staining with cytoplasmatic membrane reinforcement and without nuclear staining [47]. Another work recently published by Singh et al. provided supporting observations that **β*1-Catenin*-activating mutations did not contribute to ERMS tumorigenesis [48]. They found the *Wnt/*β*1-Catenin* signalling pathway to be inhibited in an ERMS cell line derived from *p53/c-fos* double-mutant mouse tumours. Although these cells showed higher expression of *Wnt2*, *Wnt10a,* and *Wnt8b* compared to normal myoblasts, the most highly overexpressed genes were *Wnt* pathway inhibitors such as *sFRP2*, *sFRP4*, *Dkk1,* and *Nkd1*. *Wnt* receptors (*Frizzled 1*, *3,* and *5*), the signalling mediator *Dvl* and factors involved in recruiting and forming the activation complex with **β*1-Catenin* in the nucleus (*LEF-1 and Pygo*) were downregulated. Moreover, the majority of downstream target genes of this pathway showed no expression differences compared to normal myoblasts, thereby suggesting absence of oncogenic activation of the pathway. On the other hand, some *Wnt* genes involved in myogenesis showed an expression pattern that may lead to impaired muscular differentiation. Thus, those authors reported a down-regulation of *Wnt7b*, which promotes myogenic differentiation, and up-regulation of 2 proteins, *sFRP2* and *Wnt2*, which inhibit myogenic differentiation. Furthermore, the activation of the *Wnt* pathway raised *MyoD* and *MyHC* expression levels and promoted myoblast fusion, a fact confirming that *Wnt* signalling directly promotes myogenic differentiation [48]. Taken together, the results of these experiments suggest that the activation of *Wnt* pathway in RMS may mainly promote antioncogenic effects.

## 5. *Notch*, *Wnt*, and *Hedgehog* Compensative Cross-Talking

A general drawback when pharmacological pathway inhibition is attempted is that only moderate decreases in the expression of downstream targets are achieved. This observation is often attributed to incomplete pathway inhibition by the drugs or compounds used. However, deeper understanding of the complex nature of cells and their adeptness at rewiring molecular circuitry to evade target-specific agents may permit the identification of new molecular targets and lead to the development of novel and more powerful therapeutic approaches.

In support of this idea, Ingram et al. recently reported in mesodermal and neural cells that *Hes1* transcription can also be activated by *Hedgehog* ligands by a mechanism that is absolutely independent of *Notch* pathway signalling [49]. Therefore, this mechanism of *Hes1* activation bypasses **γ*-secretase* inhibition—or any other kind of *Notch* inhibition—and may maintain significant *Hes1* expression even in the complete absence of *Notch* activation. Moreover, the *Notch* target *Hes1* can directly bind the *Gli1* promoter by acting as a transcriptional repressor and may therefore influence *Hh* signalling in glioblastoma [50]. Interestingly, that work suggested that *γ*-secretase-mediated *Notch* inhibition may lead to a rise in *Gli1* levels which may produce alterations in *Hh *signalling that in turn may promote tumour survival by *Hh* overactivation.

The *Notch* ligand *Jagged1* has also been described as a link between the *Notch* and *Wnt* pathways. The first evidence reported stemmed from an *in silico* phylogenetic analysis by which the *Jagged1* promoter was identified as a conserved target of *Wnt/*β*-Catenin* signalling based on the conservation of specific consensus binding sites [51]. Three years later, Rodilla et al. showed *Jagged1* to be the pathological link between the *Wnt *and *Notch* pathways in colorectal cancer [52]. Hence, that work established that *Notch *can be a downstream target of *Wnt* via **β*-Catenin*-mediated transcriptional activation of the *Notch* ligand *Jagged1*. Other *Wnt-*pathway-belonging proteins such as *Dvl* and *GSK3*β**have also proved to be involved in a cross-talking with *Notch*. *GSK3*β**directly binds to the *Notch2* ankyrin repeats in the HEK-293 (human embryonic kidney 293) cell line [53] while *Dvl* binds *Notch2* within its C-terminal region in *Drosophila* [54]. In both cases, *Notch* activity was reduced. The capability of **β*-Catenin* to modulate the level and transcriptional activity of *Notch1/NICD* through its direct interaction has also been described in HEK-293 cell line [55].

**Figure 1:**
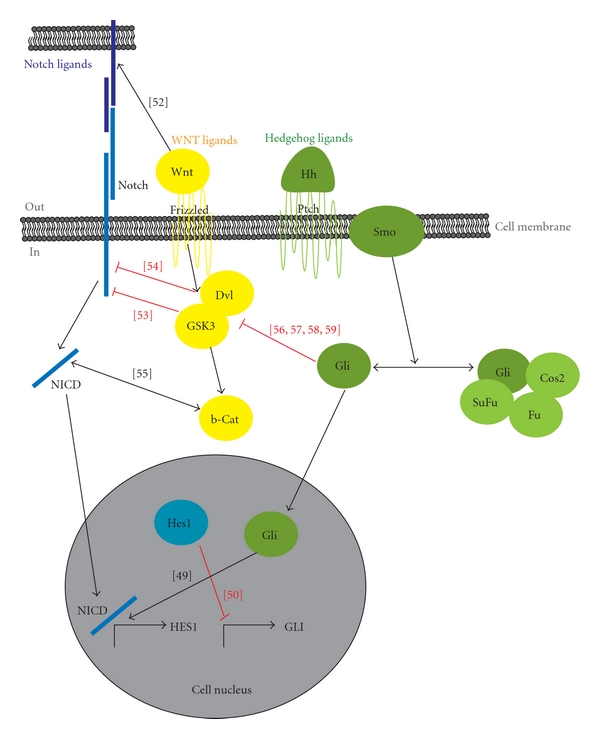
Schematic simplified view of *Notch* (blue), *Wnt* (yellow), and *Hedgehog* (green) signalling pathways and the most significant horizontal interactions among them. Black arrows indicate activating interactions. Red lines indicate inhibiting interactions.

Finally, the ability of the *Hedgehog* pathway to modulate *Wnt* signalling has also been reported. The negative regulation of *Wnt* signalling by *Gli3R* activity has been described in mouse and chick embryos [56]. Furthermore, Yanai et al. showed that *Gli1* overexpression suppressed *Wnt *transcriptional activation and found an inverse correlation between *Wnt* and *Hh* activation in human gastric tumours [57]. Additionally, the ectopic expression of *Gli1* increased the levels of *secreted Frizzled-related protein-1* (*sFRP1*) by direct binding to its promoter in gastric cancer cells [58]. In colorectal cancer, *Gli1* was shown to inhibit the proliferation of cancer cells by suppressing activation of the *Wnt* signalling pathway [59]. The main interactions described in this section for the three pathways are summarised in Figure 1.

In *Notchγ*-secretase-mediated inhibition, we observed that the reduction in *Hes1* protein levels in RMS cells *in vitro* after GSI treatment was only moderate [18]. Likewise, the reduction in *Hes1* in a RMS xenograft mouse model treated with *γ*-secretase inhibitors was also low. The low inhibition achieved could be explained by low efficiency of the drug or concentration used. Alternatively, the remaining *Hes1* expression may be explained, at least in part, in terms of compensatory mechanisms based on pathway cross-talking. Similarly, several works with xenografted rhabdomyosarcoma models agree on the possibility of reducing, but not abolishing, tumour growth by *Hh* pathway inhibition [33–35]. The compensative activation of other pathways could explain this partial resistance to *Hh*-specific therapies. The current lack of knowledge on pathway cross-talking in RMS renders it impossible to substantiate these hypotheses; however, the information recently gathered on cross-talking in other tissues may provide us with the guidelines for future research in this field.

Knowledge accumulating in recent years has rendered the extensive cross-talk among signalling pathways clearly manifest. The simplistic view of the pathways as linear entities should give way to a vision of a complex network formed by longitudinal and transverse interactions as a prior step towards improvements in future pathway-targeted therapies. At least in some cancers, deeper understanding of the adeptness of cells at rewiring molecular circuitry to evade target-specific therapies should aid the development of more successful molecular therapies.
